# A Modified Hopfield Neural Network Algorithm (MHNNA) Using ALOS Image for Water Quality Mapping

**DOI:** 10.3390/ijerph13010092

**Published:** 2015-12-30

**Authors:** Ahmed Asal Kzar, Mohd Zubir Mat Jafri, Kussay N. Mutter, Saumi Syahreza

**Affiliations:** 1 School of Physics, Universiti Sains Malaysia, Penang 11800, Malaysia; mjafri@usm.my (M.Z.M.J.); kussaynm@yahoo.com (K.N.M.); ssyahreza@yahoo.co.id (S.S.); 2 Physics Department, Faculty of Science, Kufa University, Najaf 31001, Iraq; 3 Physics Department, College of Education, Al-Mustansiriya University, Baghdad 10001, Iraq; 4 Department of Physics, Syiah Kuala University, Darussalam, Banda Aceh 23111, Indonesia

**Keywords:** Hopfield neural network, remote sensing, ALOS, water quality mapping, TSS, environmental risk

## Abstract

Decreasing water pollution is a big problem in coastal waters. Coastal health of ecosystems can be affected by high concentrations of suspended sediment. In this work, a Modified Hopfield Neural Network Algorithm (MHNNA) was used with remote sensing imagery to classify the total suspended solids (TSS) concentrations in the waters of coastal Langkawi Island, Malaysia. The adopted remote sensing image is the Advanced Land Observation Satellite (ALOS) image acquired on 18 January 2010. Our modification allows the Hopfield neural network to convert and classify color satellite images. The samples were collected from the study area simultaneously with the acquiring of satellite imagery. The sample locations were determined using a handheld global positioning system (GPS). The TSS concentration measurements were conducted in a lab and used for validation (real data), classification, and accuracy assessments. Mapping was achieved by using the MHNNA to classify the concentrations according to their reflectance values in band 1, band 2, and band 3. The TSS map was color-coded for visual interpretation. The efficiency of the proposed algorithm was investigated by dividing the validation data into two groups. The first group was used as source samples for supervisor classification via the MHNNA. The second group was used to test the MHNNA efficiency. After mapping, the locations of the second group in the produced classes were detected. Next, the correlation coefficient (R) and root mean square error (RMSE) were calculated between the two groups, according to their corresponding locations in the classes. The MHNNA exhibited a higher R (0.977) and lower RMSE (2.887). In addition, we test the MHNNA with noise, where it proves its accuracy with noisy images over a range of noise levels. All results have been compared with a minimum distance classifier (Min-Dis). Therefore, TSS mapping of polluted water in the coastal Langkawi Island, Malaysia can be performed using the adopted MHNNA with remote sensing techniques (as based on ALOS images).

## 1. Introduction

Water is one of the most important natural resources and the lifeblood for sustaining economic development in any country. Environmental and water resource management is essential for sustaining water quality for humans and other life forms on earth [1]. Water pollutants that deteriorate water quality affect many estuarine and freshwater ecosystems on earth [2]. Water quality changes in surface water bodies can create various health problems for living creatures [3]. To solve water pollution issues, we must identify the causes and detect their locations, beginning with major pollution sources that impact places of significant interest or importance. Langkawi Island is used as our study area. The area was chosen because of its urgent need for environmental and ecosystem protection. Thus, human activities and natural influences continue to increase pollution levels. We must apply monitoring techniques, analyze water bodies, and determine pollution sources to solve pollution problems. All of these requirements can be satisfied via remote sensing techniques [4].

The use of neural networks (NNs) is an important technique in remote sensing applications. NNs help to provide several useful processes, such as recognition, classification, enhancement, analysis, estimation, and prediction. Many studies have been performed related to water quality. Among several types of NNs, the Hopfield neural network (HNN) is a simple, common, and fast network, but it has problems when it deals with color (high resolution) images. The drawback associated with using an HNN with high information, multi-color images is that information is reduced when these images are converted into black and white, which is required by HNNs. Therefore, this drawback is a challenge for high-resolution images, such as satellite images, where each pixel is considered a significant piece of information used to interpret a certain phenomenon.

In 2010 Mutter [5] modified an HNN to create the MHNN algorithm, which deals with color satellite images by slicing each band into eight binary images called bitplanes and dealing with each bitplane as an independent binary image. This algorithm was applied for water quality by adopting two classes (polluted and unpolluted water) visually. However, this work is not sufficient for water quality mapping (classification) for many reasons. Firstly, MHNN is not a feed-forward auto-associated memory that is suitable for classification purposes; it was adopted as a feed-back (recurrent) auto-associated memory that is used for applications such as enhancement and restoration, so it depended on just two classes that easily gave results but not enough to be accurate for all water classes, where it had problems related with the local minimum, resulting in error and time consumption (as well as the use of a self-connection architecture, which takes more time than a non-self-connection one). In addition, MHNN considers each bitplane to have the same level, but the levels are not the same, which causes error in the results as well. Secondly, the study did not use validation data to determine the accuracy of the algorithm. Thirdly, MHNN has never been tested with noise. According to these studies, there is a gap for using HNN for classification of satellite images for an important purpose related to water quality mapping.

The aim of this study is to modify the modified Hopfield neural network (MHNN) [5] with remote sensing image and overcome the weakness points that make this network not good for water quality mapping. The mapping by the new modification is for TSS in the waters coastal Langkawi Island, Malaysia. This study has a benefit to the scientific community through using the simplest network with remote sensing images for an important problem related with water that is essential for all living creatures.

## 2. Study Area

The location of Langkawi Island in the northwest coast of Peninsular Malaysia, near to the Thailand limits. It is located approximately at latitude 6°5′ N to 6°35′ N and longitude 99°35′ E to 99°55′ E. it has an area of 362 km^2^, Langkawi Island consists mainly of forest (52%), agricultural land (18%), rubber plantations (12%), mangroves (11%), and newly expanded urban areas (5%) [6,7]. The island has become a desired destination for both local and international tourists in the last decade, although the sources of pollutant accumulation by the industrial and agricultural activities in the island’s aquatic environment [8]. The estuarine areas of Langkawi Island and the coastal are active sites where a large amount of sediment goes into the ocean system via rivers, terrestrial runoffs, and leachates carrying chemicals originating from numerous urban places [9]. This extensive type of development directly affects the marine ecosystem and causes pollution in the coastal and nearby areas [10]. The above reasons made us adopt this area, so, we adopted the west part of Langkawi Island’s coastal waters which is the most important part.

The adopted satellite in this work is ALOS. It gives classification maps for monitoring regional environments and spatial land-coverage maps [11]. Table 1 and Table 2 shows the technical specifications and characteristics of ALOS respectively [7].

**Table 1:** Technical specifications of ALOS.

Number of Bands	4	Range
Wavelength	Band 1 (blue)	420–500 nm
	Band 2 (green)	520–600 nm
	Band 3 (red)	610–690 nm
	Band 4 (near infrared)	760–890 nm
Spatial resolution	10 m	
Swath width	70 km	

**Table 2:** ALOS Characteristics.

ALOS Index	
Launch Date	24 January 2006
Launch Vehicle	H-IIA
Launch Site	Tanegashima Space Center
Spacecraft Mass	Approx. 4 tons
Generated Power	Approx. 7 kW (at End of Life)
Design Life	3–5 years
Orbit	Sun-synchronous sub-recurrent
	Repeat Cycle:46 days Sub Cycle: 2 days
	Altitude:691.65 km (at equator)
	Inclination: 98.16 deg.
Attitude Determination	2.0 × 10.4 degree (with GCP)
Position Determination	1 m (off-line)
Date Rate	240 Mbps (via Date Relay Technology Satellite) 120 Mbps (Direct Transmission)
Onboard Data Recorder	Solid-state date recorder (90 Gbytes)

## 3. Methodology

It is important to note the architecture of the MHNNA [12], this architecture consists of three neurons, where each neuron is connected with every other neuron but not to itself, for that the adopted architecture called non-self-architecture (zero diagonal weight matrixes). The possible number of vectors is $2^{3}$, the small number of neurons make the process of MHNNA computing faster than other sizes, in addition to the ability of using a big number of patterns.

In this study, the MHNN technique has been developed for mapping TSS water pollutant concentrations. Depending on the modification technique, each band of the adaptive HNN image will be sliced into binary bitplanes [5,12,13]. Each band in the satellite image is converted to eight bitplanes by converting each band value in the pixel from decimal to binary, then to a bipolar system, which is convenient for HNNs. The MHNNA consists of two phases: a Learning phase and Converging phase.

### 3.1. Learning Phase

This phase is related with the network vector which is called the known vector, and the learning weight. The adopted vector of this network consists of three elements, which are extracted from one band. The sample vectors are considered known vectors and are used for initializing the learning weights according Equation (1). This means the weight will be a matrix of nine elements. The weight initializing occurs in the learning phase of this network, and these weights are stored in a look-up table until the converging phase of the HNN with the unknown vectors, which also consist of three elements from the image data. The weight equation is called the Hebb rule [5,14,15].

(1) $$W_{ij}=\overset{3}{\underset{i,j=1}{\sum }}V_{i}V_{j\;}$$

Then, it is multiplied by the majority description of the known vector $mdkv\left(V_{j}\right):$

(2) $$W_{ij}=\overset{3}{\underset{i,j=1}{\sum }}V_{i}V_{j\;}*mdkv\left(V_{j}\right)$$

where $V_{j\;}$ is the known vector and $V_{i}$ is the transpose of $V_{j\;}$.

(3) $$mdkv\left(v_{j}\right)=sgn\left(\overset{3}{\underset{j=1}{\sum }}v_{j}\right)$$

In addition, the initialized weight has been determined with zero-diagonal architecture (non-self-connection architecture). The majority description is very important for avoiding similar weights produced by the orthogonality phenomenon. Table 3 shows the possible cases of the adopted vector and its weights, with the correction by $mdkv\left(V_{j}\right)$ [5].

**Table 3:** All the possible vectors with their weight matrices.

Index	Vector States (Binary)	Vector States (Bipolar)	Learning Weight States (Bipolar)	Majority Description $mdkv\left(V_{j}\right)$	Corrected Weight States
0	0, 0, 0	−1, −1, −1	$\left[\;\begin{array}{ccc} 0 & \;1 & \;1\;\\ 1 & \;0 & \;1\;\\ 1 & \;1 & \;0\; \end{array}\right]$	−1	$\left[\begin{array}{ccc} \;0 & -1 & -1\\ -1 & \;0 & -1\\ -1 & -1 & \;0 \end{array}\right]$
1	0, 0, 1	−1, −1, +1	$\left[\begin{array}{ccc} \;0 & \;1 & -1\\ \;1 & \;0 & -1\\ -1 & -1 & \;0 \end{array}\right]$	−1	$\left[\begin{array}{ccc} \;0 & -1 & \;1\\ -1 & \;0 & \;1\\ \;1 & \;1 & \;0 \end{array}\right]$
2	0, 1, 0	−1, +1, −1	$\left[\begin{array}{ccc} \;0 & -1 & \;1\\ -1 & \;0 & -1\\ \;1 & -1 & \;0 \end{array}\right]$	−1	$\left[\begin{array}{ccc} \;0 & \;1 & -1\\ \;1 & \;0 & \;1\\ -1 & \;1 & \;0 \end{array}\right]$
3	0, 1, 1	−1, +1, +1	$\left[\begin{array}{ccc} \;0 & -1 & -1\\ -1 & \;0 & \;1\\ -1 & \;1 & \;0 \end{array}\right]$	+1	$\left[\begin{array}{ccc} \;0 & -1 & -1\\ -1 & \;0 & \;1\\ -1 & \;1 & \;0 \end{array}\right]$
4	1, 0, 0	+1, −1, −1	$\left[\begin{array}{ccc} \;0 & -1 & -1\\ -1 & \;0 & \;1\\ -1 & \;1 & \;0 \end{array}\right]$	−1	$\left[\;\begin{array}{ccc} 0 & \;1 & \;1\\ 1 & \;0 & -1\\ 1 & -1 & \;0 \end{array}\;\right]$
5	1, 0, 1	+1, −1, +1	$\left[\begin{array}{ccc} \;0 & -1 & \;1\\ -1 & \;0 & -1\\ \;1 & -1 & \;0 \end{array}\right]$	+1	$\left[\begin{array}{ccc} \;0 & -1 & \;1\\ -1 & \;0 & -1\\ \;1 & -1 & \;0 \end{array}\right]$
6	1, 1, 0	+1, +1, −1	$\left[\begin{array}{ccc} \;0 & \;1 & -1\\ \;1 & \;0 & -1\\ -1 & -1 & \;0 \end{array}\right]$	+1	$\left[\begin{array}{ccc} \;0 & \;1 & -1\\ \;1 & \;0 & -1\\ -1 & -1 & \;0 \end{array}\right]$
7	1, 1, 1	+1, +1, +1	$\left[\begin{array}{ccc} \;0 & \;1 & \;1\;\\ \;1 & \;0 & \;1\\ \;1 & \;1 & \;0 \end{array}\;\right]$	+1	$\left[\begin{array}{ccc} \;0 & \;1 & \;1\;\\ \;1 & \;0 & \;1\;\\ \;1 & \;1 & \;0\; \end{array}\right]$

### 3.2. Converging Phase

The convergence phase of this network is represented by the energy function equation. It gives the value that denotes the amount of convergence between the known vector (from the sample), which is represented by the learning weight, and the unknown vector (from the image). The energy function of the Hopfield neural network is given by the following equation [16]:

(4) $$E=-\;\frac{1}{2}\;\overset{n}{\underset{i}{\sum }}\overset{n}{\underset{j}{\sum }}v_{i}w_{ij}v_{j\;}+\delta \;v_{j}$$

where *E* = energy function, *n* = number of vector elements, $v_{j\;}$ = unknown vector = ${\text{v}}_{i}^{T}$ or $v_{i}$ = ${\text{v}}_{j}^{T},\;Wij$ = learning weight of the known vector which is the counterpart of the unknown vector ${\text{v}}_{j}$, where this weight from the output of neuron *i* to the input of neuron *j* and δ = the limiting value, which equals zero in the Hopfield neural network because it is a single layer.

Because δ = 0, the energy function equation becomes:

(5) $$E=-\;\frac{1}{2}\;\overset{n}{\underset{i}{\sum }}\overset{n}{\underset{j}{\sum }}v_{i}w_{ij}v_{j\;}$$

In the MHNNA, the energy function equation has been modified by considering weights for the bitplanes (Wb) to keep the energy function level of each of the eight bitplanes. Additionally, the energy function must be multiplied by the majority description of the unknown vector $mduv\left(v_{j}\right)$, to avoid yielding the reverse result for the energy function [12]. Therefore, the equation for *E* becomes:

(6) $$E=-\;\frac{1}{2}\;\overset{n}{\underset{i}{\sum }}\overset{n}{\underset{j}{\sum }}v_{i}w_{ij}v_{j\;}*Wb*\;mduv\left(v_{j}\right)$$

(7) $$mduv\left(v_{j}\right)=sgn\left(\overset{3}{\underset{j=1}{\sum }}v_{j}\right)$$

The weight of a bitplane (*Wb*) is represented by the following equation:

(8) $$Wb=2^{L-1}$$

where *Wb* is the weight of a bitplane in the binary system and *L* is the bitplane order [4] so that, without this weight, the energy function will be equal in the eight levels without distinguishing properly of the differences and the similarities between the samples pixels and the image pixels. But by considering *Wb* the differences and the similarities will be distinguished easily and properly for classification purpose by MHNNA. Where the physical meaning relates with these equations is represented the quantum value of the reflected radiation from water body which is received by the satellite sensor, so the job of MHNNA is to find the convergence between the quantum values of the samples and the image pixels via the energy function which represented by Equation (6) all this is for classification purposes of the concentrations of water pollutant (TSS). Table 4 gives *Wb* values for each bitplane order in the binary system.

**Table 4:** Bitplane orders with their weights in the binary system.

*L*	1	2	3	4	5	6	7	8
*Wb*	1	2	4	8	16	32	64	128

Modification of the MHNNA was conducted via the following contributions.

### 3.3. Validation Data

The validation data (real data) representing TSS concentrations are divided into two groups. The first group is used to classify samples (source samples) through the converted band 1, band 2, and band 3 values of these samples locations for using in the MHNNA. The second group is used as test samples by detecting their position in the produced classes. The band values are converted from decimal to binary, then to a bipolar system as known vectors. They produce the learning weights in the learning phase of the MHNNA. The weights will then combine with the unknown vectors from the image bands to test the corresponding or nearest class [4]. The concentration values will not be used in the proposed algorithm, but instead of determining accuracy through calculating the R and RMSE between the concentrations from the produced classes and those detected in the produced class locations. Previous studies [5] have used only two samples. In this study, 13 real samples were used. They provide a better range of samples, which is ideal for satellite image classification success and algorithm accuracy. Matching then occurred between the image pixel vector and the weight of the sample vector. This matching is an important step in calculating the energy function value.

**Figure 1:**
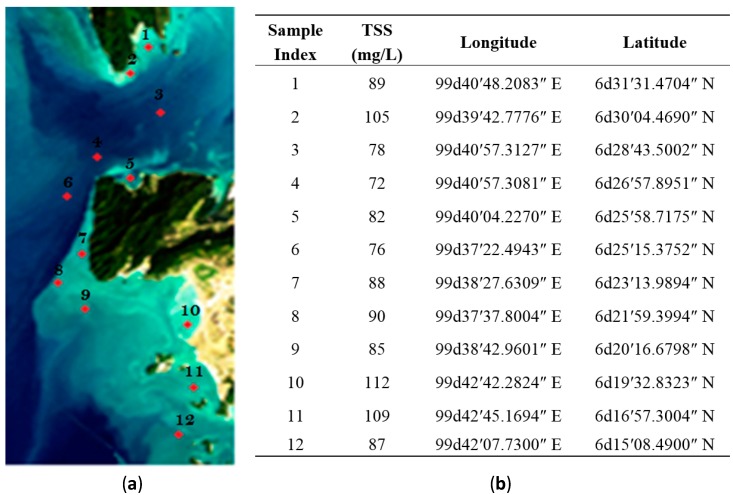
ALOS image of study area and validation data: (**a**) Raw satellite image and sampling indices; (**b**) Samples locations and their concentrations values.

The sample collection locations (longitudes and latitudes) in the study area were determined using a handheld global positioning system (GPS). Samples from these locations are collected simultaneously with satellite (ALOS on 18 January 2010) image acquisition. The samples were analyzed in the lab to measure the validation data, represented by TSS concentrations in milligrams per litre (mg/L) for each location. The raw satellite imagery of the study area and sampling locations that are used for validation data and the TSS concentrations with indices are shown in Figure 1.

### 3.4. MHNNA as a Feed-Forward Associative Memory

The adopted HNN in this study is represented as a feed-forward network, which acts in one direction. It is not recurrent because this network has been determined as ideal for classification purposes. Two types of convergence methods exist [12,14]:

Type One: Detection method—used to detect the corresponding vector via calculating the value of the energy function. This method is related to a feed-forward net (non-iterative net) and is typically used for pattern classification.

Type Two: Generating method—used to generate the lost vector depending on the energy function value calculation. This method is a feed-back net (iterative net) and is typically used for enhancement or restoring purposes.

We have adopted a detection method using satellite (ALOS) images to classify pollutants and contribute to water quality mapping. MHNNA without iterations in the converging phase provides two advantages: (1) The network will not face local minimum problems, which produce errors in the results; and (2) It will increase algorithm speed, *i.e.*, reduce the algorithm run time.

In addition, because [5] used an HNN generating method for dealing and optimizing colour images and we used the HNN for dealing with and classifying colour images, a comparison will be made between the minimum distance classifiers, which have been used to classify satellite images in many previous studies [17,18].

### 3.5. Considering Bitplane Weights in the Energy Function

The binary number value in the bitplane increases as the order increases from lowest to highest. Without Wb, the energy function value of a bitplane will be based on the nearest or similar value of the energy function of another bitplane, without considering the binary number level of the bitplane. Therefore, each bitplane has a differing effect on the summation value of the eight energy functions. The selected sample (class) for image pixels, as a result of classification, is the sample that gives the lowest summation of energy functions because the Hopfield neural network denotes the corresponding or nearest class. The Wb in the MHNNA is very important for correcting the results. This variable avoids summation error in the energy functions values.

Thus, we have modified the HNN, a simple network, to utilize modern techniques (satellite imagery) to analyze an important topic, water pollution. So, the bands used in this study are three: band 1, band 2, and band 3 that represent blue, green, and red respectively. Where these bands are given via reflectance values and each band has contributed by a certain ratio according to the Table 5. These ratios gave the best results of using MHNNA with ALOS 18 January 2010 image.

**Table 5:** The adopted ratios of used bands for TSS mapping by ALOS 18 January 2010 image.

Band	Ratio
Band 1 (blue)	0.7
Band 2 (green)	0.2
Band 3 (red)	0.1

## 4. MHNNA

The new algorithm is represented as the following:

(1). Collect samples as validation data (*in-situ* data) from the study area simultaneously with the acquisition of the satellite imagery, fixing the position of these data via a handheld global positioning system (GPS).
(2). Determine the pollutant concentration values of the collected samples in the lab.
(3). Order the pollutant concentration values in ascending order. Then, separate them into two groups. The first group includes values that have odd indexes, which are used as classes for supervised classification. The second group includes values that have even indexes, which are used to validate the algorithm accuracy.
(4). Use the satellite image and give the non-water pixels a black color (band 1 = 0, band 2 = 0, band 3 = 0).
(5). Learning phase: for water pixels, analyze each pixel of the bands and multiply each band by its ratio through Table 5. Then for each band:
(6). Convert each digital number from decimal to binary representation (eight bits), *i.e.*, replacing the band with eight binary bitplanes (eight binary images). Each pixel in a bitplane will be 0 or 1. Note that for small binary values (e.g., 3 is equal to binary 11), the remaining six bits (bitplane) to the left will have zero values (e.g., 00000011).
(7). Convert the bitplane numbers from binary to bipolar representation by considering each (0) to be (−1) and each (1) to be (+1).
(8). Choose 3 × 3 pixel × 8 bitplane sample sizes for each image band, which represent the samples for supervised classification. The locations of these samples are the exact locations (longitudes and latitudes) of collected samples (real data) from the study area.
(9). Initialize 1 × 3 vectors by dividing each sample size in each band in each bitplane by the 1 × 3 size (*i.e.*, each chosen sample size will have 3 vectors) to be included in the known (learning) vector.
(10). For each known vector, find the learning weight via Equation (2) and depending on the vector information. These weights are given sequentially symbols and saved in the lookup table:
  (9) $$W_{ij}^{sbpc}=\overset{n}{\underset{i}{\sum }}\overset{n}{\underset{j}{\sum }}V_{i}^{sbpc}.V_{j}^{sbpc}\;md\left(V_{j}^{sbpc}\right)$$
(11). Where **s** is number of samples in band **b** in bitplane **p** of number of known vector **c**.
(12). Converging phase: Starting from the beginning of the water pixels in image I, for each band b and each bitplane p, obtain a 3 × 3 sample size, then divided these samples into three 1 × 3 vectors, which will be unknown vectors: $V_{j}^{Ibpc}$
(13). Find the energy function values for each vector in the first sample and its counterpart vector from the image, as shown in:
  (10) $$E^{bpc}=-\;\frac{1}{2}\;\overset{n}{\underset{i}{\sum }}\overset{n}{\underset{j}{\sum }}V_{i}^{Ibpc}W_{ij}^{sbpc}\;V_{j}^{Ibpc}\;Wb^{p}\;md\left(V_{j}^{Ibpc}\right)$$
(14). Calculate the Energy Function summation values for the eight bitplanes of each band. Then, find the SEF value for the three bands (band 1, band 2, and band 3) related convergence of the first sample.
  (11) $$SEF=E^{b}+E^{g}+E^{r}$$
  where $E^{b}$, $E^{g},and\;E^{r}$ are the Energy Function summation values for the band 1, band 2, and band 3, respectively.
(15). Repeat 13 and 14 for all other samples.
(16). Find the minimum summation value (SEF) from all summations values. Then, note the image size of the sample (class) that produced this minimum value.
(17). Genrate a classified image by giving color for each class to be color coded.
(18). Validate the results through identifying the class values that cover the location of the second group values (e.g., each value that represents its class and the value from the second group which found in this class). Then, find the correlation coefficient, R, and RMSE between them.

## 5. Results and Discussion

**Figure 2:**
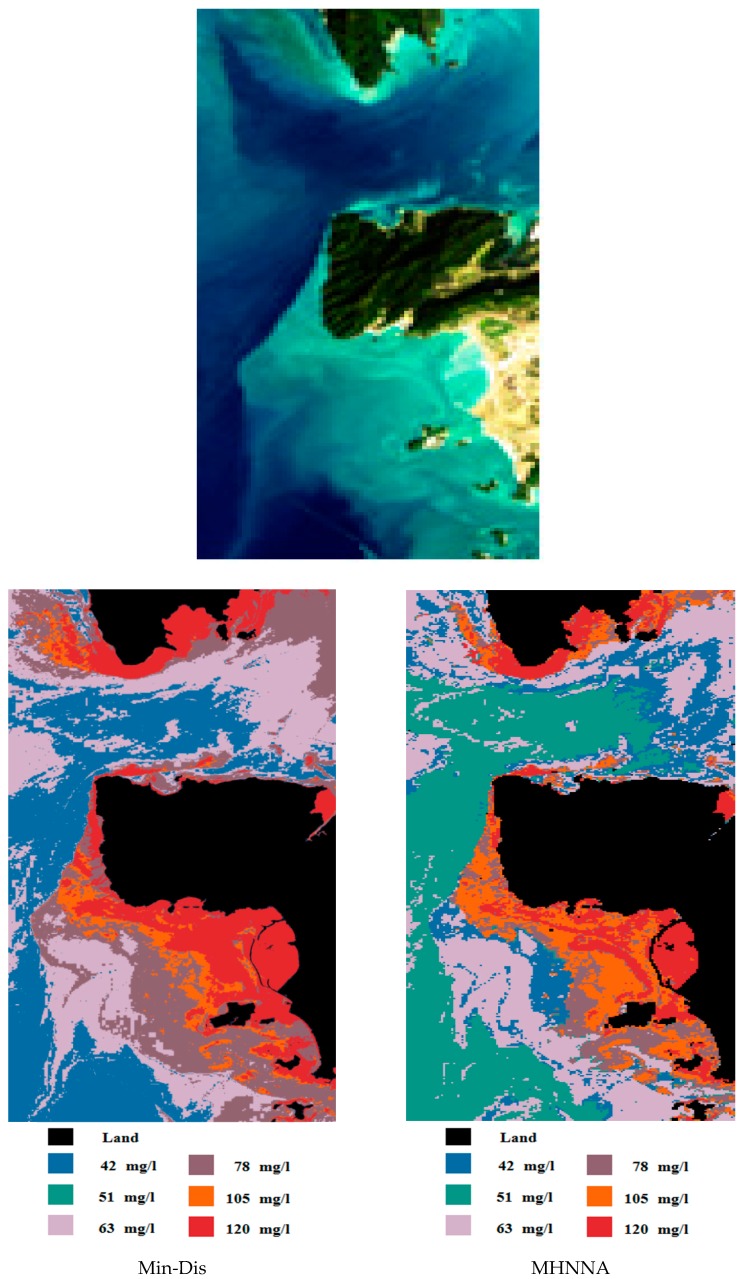
TSS mapping using the MHNNA compared with a Min-Dis classifier using the ALOS 18 January 2010 image.

The results obtained by applying the proposed algorithm to the adaptive satellite image are shown in Figure 2.

Via visual evaluation, we find that the first class in the MHNNA map is more homogenous than the first class in the Min-Dis map. In addition, there are multiple differences between the two maps, but a visual evaluation is not sufficient for synoptic comparison. Therefore, the following two tables provide a quantification of the differences (Table 6 and Table 7).

**Table 6:** The sample accuracies of the classes produced by MHNNA and Min-Dis.

Min-Dis		MHNNA	
Classification Samples (mg/L)	Test Samples (mg/L)	Classification Samples (mg/L)	Test Samples (mg/L)
72	76	78	76
88	82	78	82
85	87	85	87
109	89	88	89
109	105	109	105
109	112	109	112

**Table 7:** Accuracy results.

	R	RMSE
MHNNA	0.977	2.887
Min-Dis	0.825	8.954

Table 6 denotes the concentration values of the classes produced via each method. Although the majority of the test samples are located in the correct classes, some were inaccurately classified, particularly within the Min-Dis results. This proves that the proposed algorithm is better than Min-Dis, because three classes by MHNNA gives the nearest values comparing with the Min-Dis, where these classes are the first, second, and fourth that represent by 78, 78, and 88 respectively. The MHNNA is capable of high quality TSS mapping (classification). The minimum distance classifier (Min-Dis) has limitations related with affecting the extrema values on the mean value [19], but the new algorithm (MHNNA) has strength related with dealing deeply with numbers through the eight bitplanes without affecting the extrema values.

Regarding the efficiency of the proposed algorithm, we calculated the correlation coefficient (R) and the root-mean-square-error (RMSE) between the first and the second groups of real data. Table 7 displays the accuracy results.

Higher R and lower RMSE related to the ability of the method that related more classes for the image values correctly. For this, the results in this table provide evidence that MHNNA yields a more accurate result than the Min-Dis. This is related to producing classes properly.

## 6. Noise Test

This type of images, which contains TSS pollutant information, has also been tested for noise. Salt and pepper noise were added over a range of noise levels (0.01–0.3), which increased by (0.01). We then applied the MHNNA and Min-Dis methods for producing maps and calculated the accuracies for each noise level.

**Figure 3:**
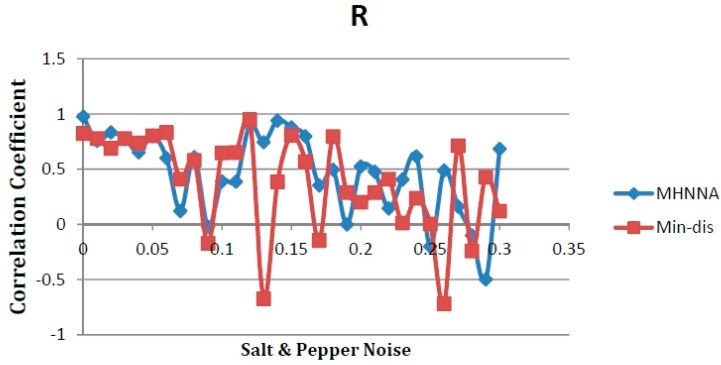
Testing the MHNNA with salt and pepper noise and comparison with the Min-Dis method, as applied to an ALOS image of TSS concentrations, with accuracy evaluated via a correlation coefficient (R).

Figure 3 illustrates that the new MHNNA proved to be consistently accurate when coping with various noise levels. Conversely, the Min-Dis method decreased in accuracy for most noise levels. Bottoms and tops were produced via both methods, especially when using the Min-Dis method. This is because the added noise produced additional band values randomly, which affected classification accuracy, producing bottoms. In addition, increasing the sample band values supports the learning phase and increases the image band values that support the convergence phase. This leads to increased convergence, thereby increasing the accuracy and producing tops in the noise test curves. However, the results provide evidence that the proposed algorithm can give accuracy even with noise, but for comparing we could not determine which one is better, therefore we calculate the mean values of all correlation coefficients with all noise quantities and for the both method as in the Table 8.

**Figure 4:**
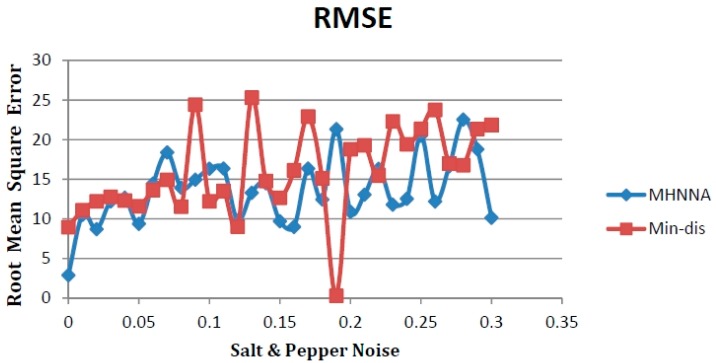
Testing the MHNNA with salt and pepper noise and comparison with the Min-Dis method, as applied, to an ALOS image of TSS concentrations, with accuracy evaluated via a root mean square error (RMSE).

Figure 4 is similar to Figure 3, further suggesting that the MHNNA can successfully cope with various noise levels. Therefore, we calculated the mean values of both accuracy measurements across all of the noise quantities, yielding synoptic measurements for each comparison method for giving the decision about which one better fares against noise. The results are shown in Table 8.

**Figure 5:**
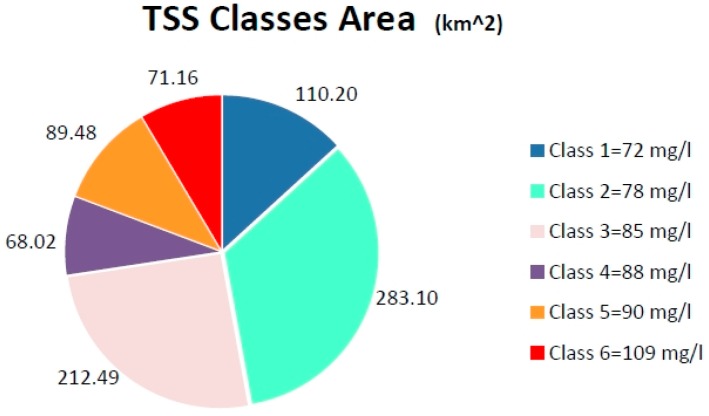
The areas of the TSS concentrations classes.

After proving the success of the proposed algorithm for water quality by using a remote sensing image, providing more information about the environmental health in this area, we calculated the areas of each class (each TSS concentration level) as depicted in Figure 5.

**Table 8:** R and RMSE means of applying MHNNA and Min-Dis methods with noise.

	Mean of R	Mean of RMSE (mg/L)
MHNNA	0.490	13.624
Min-Dis	0.388	15.889

These several areas of TSS classes (concentrations) give the synoptic measurements about this pollutant, where the biggest area related with the Class 2 has concentration of 78 mg/L that is lower than other classes except for class 1, indicating that it is less dangerous. This shows comfortable environmental information about water quality within the area. However it is disturbing that classes 5 and 6 with concentrations of 90 mg/L and 109 mg/L respectively are not only larger than the least polluted area (class 4 = 88 mg/L) but lie very close to the island, creating high risk in water quality, which results in intensive TSS concentrations in the area.

## 7. Conclusions

MHNNA exhibited a higher R (0.977) and lower RMSE (2.887). Also, the testing of the new algorithm with noise proves its accuracy with noisy images over a range of noise levels comparing with the minimum distance classifier (Min-Dis). This study provides a brief overview of TSS mapping at the Langkawi Island, Malaysia. Satellite imagery can be used to provide information for effective planning management. Via MHNNA, we get extra information about the pollutants related with the areas of the TSS concentrations classes. Applying the MHNNA to ALOS data for TSS mapping in the study area produced reliable, high quality results. Our results exhibit successful evidence of applying the new algorithm to the water quality mapping of color images, which is considered a new application of HNNs, also, the produced map gives dilution of the TSS in water through the ability of finding the little differences between the band numbers of water pixels in the adopted image. Concerning the environmental health, the west side of Langkawi Island poses a water quality risk.
